# Long-term results after Boston brace treatment in adolescent idiopathic scoliosis

**DOI:** 10.1186/1748-7161-4-17

**Published:** 2009-08-26

**Authors:** Johan Emil Lange, Harald Steen, Jens Ivar Brox

**Affiliations:** 1 Orthopaedic Department, Rikshospitalet, Oslo University Hospital, Norway

## Abstract

**Background:**

Few studies have evaluated long-term outcome after bracing using validated health related quality of life outcome measures. The aim of the present study was to evaluate the long-term outcome in adolescent idiopathic scoliosis (AIS) 12 years or more after treatment with the Boston brace.

**Methods:**

109 (80%) of 135 patients (7 men) with AIS treated with the Boston brace at a mean of 19.2 (range 12–28) years previously responded to long-term follow-up examination.

All patients (n = 109) answered a standardised questionnaire including demographics, work status, treatment, Global Back Disability Question, Oswestry Disability Index (ODI) (100-worst possible), General Function Score (GFS) (100 – worst possible), EuroQol (EQ-5D) (1 – best possible), EQ-VAS (100 – best possible)) and Scoliosis Research Society -22 (SRS – 22) (5 – best possible). Clinical and radiological examination was obtained in 86 patients.

**Results:**

The magnitude of the primary prebrace major curve was in average 33.4° (range 20 – 52). At weaning and at the last follow-up the corresponding values were 28.3° (9–56) and 34.2° (8 – 87), respectively. The mean age at follow-up was 35 (27 – 46) years. Work status was: full time (80%), on sick-leave (3%), on rehabilitation (4%), disability pension (4%), homemaker (7%), students (2%), 7% had changed their job because of back pain. 88% had had delivered a baby, 55% of them had pain in pregnancy. Global back status was excellent or good in 81%. The mean (standard deviation) ODI was 6.4 (9.8), GFS 5.4 (10.5), EQ-5D 0.84 (0.2), SRS-22: pain 4.2 (0.8), mental health 4.2 (0.7), self-image 3.9 (0.7), function 4.1 (0.6), satisfaction with treatment 3.7 (1.0). 28% had taken physiotherapy for back pain the last year and 12% had visited a doctor.

**Conclusion:**

Long-term results were satisfactory in most patients with AIS treated with the Boston brace.

## Background

In a prospective study Nachemson and Peterson showed that bracing alters the natural history of adolescent idiopathic scoliosis (AIS) in the short term [1], but its efficacy in the long term has remained controversial [2]. A recently published systematic review concluded that bracing AIS is effective in the long-term [3]. Most studies evaluated long-term results in patients, who had used the Milwaukee brace, and commonly reduction of the original curve was shown at brace discontinuation with progression after treatment equivalent to the natural history. Health Related Quality of Life (HRQL) measures were comparable to that of controls, with slight differences regarding back pain and some physical activities. Another recently published systematic review reported that the percentage of brace treated patients with later surgery ranges from 1% to 43%[4]. Danielsson and Nachemson have published the most comprehensive studies including HRQL measures 22 years after treatment [5-9]. A few of the patients included in their first studies, and all the patients in the latest published study were treated with the Boston brace [9]. Another two studies have reported results up to 10 years after Boston bracing [10,11]. None of the studies have used validated scoliosis specific questionnaires. Recently the Brace Questionnaire was developed [12]. A Norwegian translation of this questionnaire was not available for use in the present study.

The aim of the present study was to prospectively evaluate progression of the scoliotic curve, and to report HRQL using both validated scoliosis specific questionnaires and generic questionnaires in patients with adolescent idiopathic scoliosis (AIS) 10 years or more after Boston brace treatment.

## Methods

618 patients with scoliosis were treated with Boston brace at Sophies Minde Hospital (Orthopaedic Department, Rikshospitalet University Hospital) in Oslo, Norway from 1976–88. Forty (6.5%) patients had surgery. 496 patients had adolescent idiopathic scoliosis and 138 of these patients who had their last follow-up no longer than 2 years after brace weaning, were selected for follow-up. Three patients were dead, and 135 patients were invited for a standardised long-term evaluation. The indication for bracing was a major scoliotic curve > 20° with an observed progression > 5° after 4 months and Risser sign < 3. Prior to bracing standing radiographs were taken in the front and lateral projections. Patients had follow-up with clinical and radiological examination at 4 months intervals throughout the brace treatment period. Wearing of the brace was assessed by one orthopaedic surgeon (JEL) and reported as used as prescribed, irregular, or aborted. Patients were recommended to use the brace for 23 hours daily. Wearing of the brace < 20 hours daily was described as irregular. After brace weaning patients had follow-up at 6, 12, and 24 months.

A standardised form was used to obtain clinical and radiological data. Radiological measurements were performed by an orthopedic surgeon (JEL) and controlled by an experienced radiologist. Both used the Cobb method manually. Digital measurements were used at long-term follow-up. The intra-observer error for the Cobb angle was about 3° in a recent study using manual and digital measurements, and <5° in a previous study [13,14]. In the present study the measurement error was within these limits as evaluated by the reproducibility of radiographic readings of repeated measurements of all radiographs from 10 patients at regular intervals. In patients with double thoracolumbar curves the largest curve prior to bracing was defined as the major curve.

### Questionnaire

At long-term follow-up, a standardised questionnaire was mailed to the patients. The questionnaire comprised validated measures of pain, disability, quality of life and work, and questions about demographics.

The Scoliosis Research Society 22 questionnaire (SRS-22) is validated and widely used for evaluation of health-related quality of life in AIS [15,16]. A recently translated and validated Norwegian version was used in the present study [17]. The SRS-22 covers four domains (function/activity, pain, self-perceived image, mental health) each with 5 questions, and one domain (satisfaction with treatment) with 2 questions. Each item has 5 verbal response alternatives ranging from 1 (worst) to 5 (best). Results are expressed as the mean (total sum of the domain divided by the number of items answered) for each domain.

Patients rated their overall function by the Global Back Disability Question [18]. This is a single question designed to measure the patients' overall rating of their back disability today. There were five response alternatives: "excellent, none or unimportant complaints," "good, occasionally bothered by back pain," "fair, some back pain and limited function," "poor, unchanged, considerable complaints and severe disability," and "miserable, worse, not self-reliant in activities of daily living".

The Norwegian version of the original Oswestry Disability Index (ODI) (version 1.0) was used to evaluate back-specific disability [18,19]. This score has 10 questions about pain and pain-related disability in activities of daily living and social participation. The sum is calculated and presented as a percentage, wherein 0% represents no pain and disability, and 100% represents the worst pain and disability.

The General Function Score (GFS) was used to measure back-related disability in activities of daily living [20]. Patients answered nine questions using one of three alternatives: "can perform", "can perform with difficulty due to back complaints" and "cannot perform due to back complaints". The score was presented as a percentage wherein 100% represents maximum disability.

EuroQol is a generic (non-disease specific) instrument for measurement of quality of life that was specifically developed for the derivation of quality-adjusted life-years (QALYs) [21-23]. The questionnaire includes five items regarding quality of daily life, covering the domains of mobility, self-care, usual activities, pain and discomfort, and anxiety and depression (EQ-5D) and a visual analogue score for assessment of overall current health (EQ-VAS). Items for EQ-5D use a 3-point adjectival response scale. Scores are transformed using utility weights from the general population to produce a single index, ranging from -0.59 for the worst possible health state to +1.00 for the best possible health state. Patients rate their overall current health (EQ-VAS) from 0 (worst imaginable) to 100 (best imaginable).

Evaluation of work status included questions about paid work (full-time, part-time, not working) and status if not working (on sick leave, vocational or medical rehabilitation, disability pension, unemployed, homemaker, or student) [18]. Norway has a National Social Security System that covers all inhabitants. Patients on sickness certification receive 100% benefit up to one year. Thereafter, they receive medical or vocational rehabilitation in order to reduce disability. If the patients are not able to work after years on rehabilitation they receive a disability pension with a lower benefit.

### Statistical analysis

Results are presented as means (standard deviation, range) or percentages. The normal distribution of baseline, follow-up data, and differences were checked by histograms. The success rate at maturity was calculated according to Nachemson and Peterson [1]. They defined success of treatment as an increase in the curve of less than 6° from the start of bracing. We compared HRQL measures in patients with a major curve > 45° and < 45°, respectively, using independent t-tests. This analysis was also applied to compare patients who had taken treatment the year preceding follow-up, with those who had not. The mean scores (standard deviation) in each group and the mean differences between groups (95% confidence intervals) were calculated.

## Results

109 (81%) patients, 102 (94%) women, filled in the questionnaire, and 86 (64%) had additional clinical and radiological examination at follow-up at mean 19.2 (range 102-28) years after Boston brace treatment. The mean age was 35 (27–46) years at follow-up.

Mean (standard deviation) age at start of bracing was 13.4 (1.9) years, bone age 12.9 (1.9) years, and age at menarche 13.5 years (1.2). The mean primary curve was 33.2° (range 20° to 52°) and 28.2° (9° to 53°) at brace weaning, Table 1. The mean primary curve was 33.4° (20° to 52°) and 28.3 (9° to 53°) at brace weaning in the 86 patients who had long-term radiological examination.

**Table 1:** Baseline characteristics; means (standard deviation) are given.

Characteristic	Had long-term follow-up n = 109	Did not have long-term follow-up n = 26
Age at the start of brace treatment (years)	13.4 (1.9)	13.1 (1.8)
Age at menarche (years)	13.5 (1.2)	13.3 (1.0)
Age at weaning (years)	16.2 (1.1)	16.0 (1.0)
Major curve at the start of brace treatment	33.2° (7.8)	34.6° (9.6)
Major curve at weaning	28.2° (9.0)	30.6° (9.8)

There were no differences between those who only responded to the questionnaire and those who had long-term radiographs. Baseline characteristics and curve size at weaning for the patients who did not respond to long-term follow-up, are reported in Table 1.

The brace was used as prescribed in 87% and used irregular or aborted in 6.5% of the patients, respectively. The mean reduction in primary curve size at weaning was 5.7° (6.4) in those who used the brace as prescribed, 1.4° (7.7) with irregular use, and -0.4° (5.5) in those who aborted, respectively. The success rate at maturity according to curve size progression was 76%.

**Figure 1:**
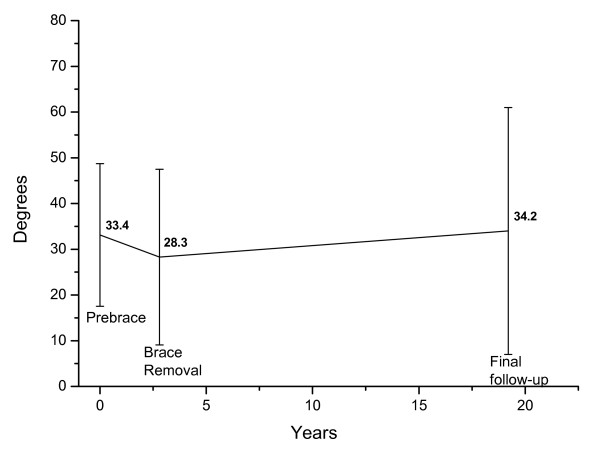
**Longitudinal development of the major curve**. Mean Cobb angle ± 2 SD prebrace, at brace removal, and at long-term follow-up in 86 patients.

The major curve size at baseline, brace weaning, and long-term are presented in figure 1. The mean primary curve was 34.2° (range 8° to 87°) at follow-up. Eleven patients had a major curve > 45° at follow-up. Among these patients the curve exceeded 60° in six patients including 85° and 87° in two patients, respectively. The average curve progression was 22.5° in the six patients with curves > 60°.

Sociodemographic characteristics at long-term follow-up are presented in Table 2. Eleven percent was either on sick leave, rehabilitation, or disability pension. Eighty-eight (87%) women had delivered a baby. Pain was increased in 55% during pregnancy, Table 3.

**Table 2:** Sociodemographic characteristics at a mean of 19.2 years follow-up in 109 patients (percentages are given).

Characteristic	Percentage
Educational level	
Primary school (9 year)	14
High school (12 year)	28
University college	58
Work status	
Working Student	80 2
Homemaker	7
On sick leave	3
Rehabilitation	4
Disability pension	4
Changed job because of back pain or disability	7
Scoliosis influenced my choice of education and job	22
Comorbidity	26
Smoking	21
Married/living together	77
Born children (n = 102)	87
Pain in pregnancy (n = 88)	55

**Table 3:** Outcome at a mean (SD) of 19.2 years follow-up in 109 patients.

Outcome	Percentage or mean (95%CI)
Global Back Question	
Excellent	25
Good	56
Fair	14
Poor	5
General Function Score (0–100)	5.4 (10.5)
Oswestry Disability Index (0–100)	6.4 (9.8)
EQ – 5D (-0.5 to 1.0)	0.84 (0.19)
EQ – VAS (0–100)	77.2 (18.2)
SRS-22 (0–5)	
Pain	4.2 (0.8)
Physical function	4.2 (0.7)
Mental health	3.9 (0.7)
Self–image	4.1 (0.6)
Satisfaction	3.7 (1.0)

**Figure 2:**
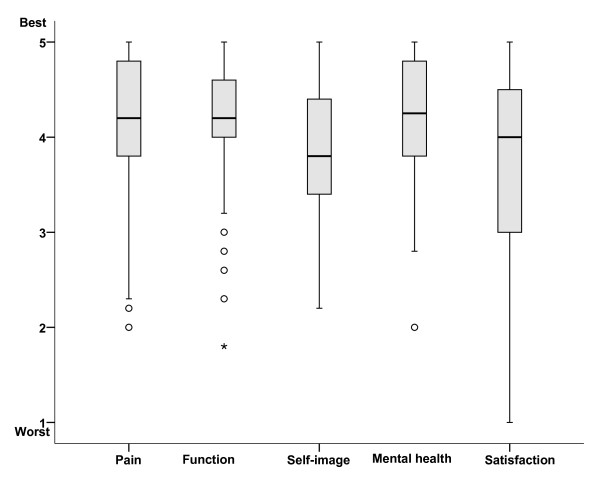
**Quality of life at long-term follow-up in AIS treated with the Boston brace**. Box-plot showing median with 25- and 75 percentiles and outliers are shown for each domain of the SRS-22.

**Figure 3:**
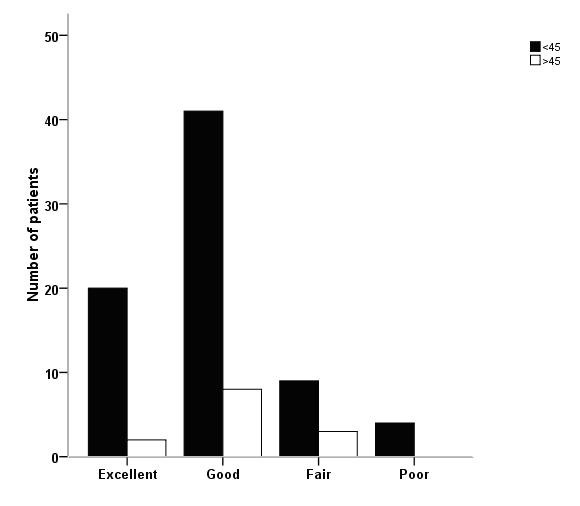
**Back function at long-term follow-up in AIS treated with the Boston brace**. Numbers are patients with scoring on the Global Back Disability Question according to major curves <45° and >45°, respectively.

**Figure 4:**
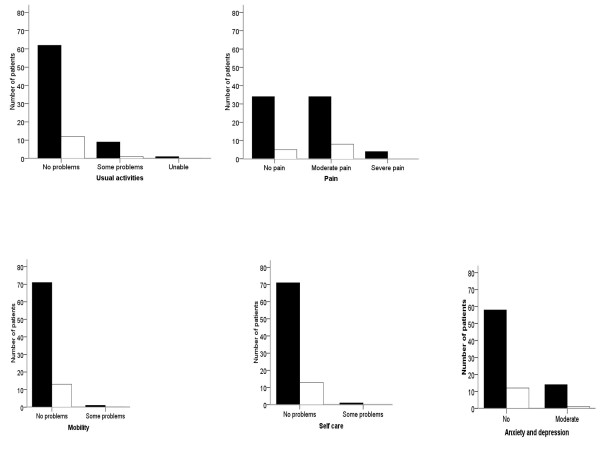
**Quality of life at long-term follow-up in AIS treated with the Boston brace**. Numbers are patients according to scoring on the five EQ-domains.

Overall back function was considered excellent, good or fair in 95% of the patients, Table 3. There was no difference between patients with curves > 45° compared with patients with curves < 45° at long-term follow-up, Figure 3. Results for the different SRS-22 domains are presented in figure 2. Scores for ODI, GFS, and EQ-5D were in the upper normal range and EQ-VAS in the normal range, Table 3. Scores for the five EQ-domains are given in Figure 4. There were no differences in patients with curves < 45° versus > 45° for any HRQL outcome measure.

Thirteen (12%) patients reported that they had consulted a physician for back complaints during the last year before follow-up, and 31 (28%) had physiotherapy. Patients, who had consulted a physician or had physiotherapy the year preceding follow-up, reported significantly higher scores on ODI, GFS, and more pain and SRS-22 subscores, compared with those who had not. No difference was observed for mental health, self-image, and satisfaction. The mean differences were 4.4 (95% confidence interval 0.7 to 8.1) for ODI, 4.7 (0.8 to 8.7) for GFS, 0.6 (0.1 to 0.4) for SRS-22-pain, and 0.2 (0.0 to 0.5) for SRS-disability. The mean scores were in the lower range for both groups, by example, 9.2 (SD 10.2) versus 4.8 (9.2) for ODI.

## Discussion

Results 19.2 years after treatment with Boston brace for adolescent idiopathic scoliosis are in agreement with previous studies on bracing [3]. Curve progression was minor in the majority of the patients. The mean curve at long-term follow-up was slightly increased when compared with the original curves and the curve after weaning. The Oswestry Disability Index and the General Function Score was slightly elevated compared with controls used in previous studies and in the same range as previously published at long-term brace treatment [5,8], and compared with results 10 years after surgical correction using Cotrel-Dubousset instrumentation [24]. The scoliosis specific scores using SRS-22 showed larger variation than the results from generic questionnaires, but were comparable with scores reported after surgery [25,26]. Recently results in 109 patients with AIS minimum 10-years after surgery using third-generation instrumentation (TSRH) were reported [27]. Mean scores ranged from 3.6 to 4.0 for the five domains. In particular, the mean score for self-image was identical, and scores for pain, function and mental status were slightly worse, while patient satisfaction was slightly better compared with the present study.

Because the mean time to follow up was twice as high and patients were older, we expected that the number of patients, who had taken disability pension, was higher compared with previously presented results after surgery at our hospital [24]. The observed number was slightly lower. The operated patients in the previous study had lower curves at follow-up.

The present study was not randomised and did not include a control group of comparable patients and was therefore not properly designed to assess the effectiveness of bracing. We cannot exclude that the long-term results of the present study represent the natural history. Also, selection bias may contribute to results compared with previous studies that were referred above, because samples are not matched on prognostic factors. However, the success rate at maturity was in agreement with that reported by Nachemson and Peterson in the only prospective controlled study published at present [1]. Treatment with brace was associated with a 74% success rate at maturity compared with 34% and 33% for electrical stimulation and observation if success was defined as an increase in the curve of less than 6° from the time of the first roentgenogram. Despite their findings bracing remains controversial and two randomised controlled trials are currently conducted to evaluate its effectiveness [28,29].

A recent systematic review used the number of surgically treated patients as an indicator of failure of bracing [4]. The percentages reported in the included studies ranged from 1% to 43%. We were not able to give the exact percentage for the brace treated patients in the present cohort, but 6.5% of the total sample had surgery. The percentage operated depends on several factors such as the magnitude of the curve and the size of the rib hump, the opinion of the spine surgeon, and the willingness of the adolescent patient and her parents. Despite progression at long-term follow up, the 11 patients with curves > 45° reported HRQL results comparable with patients with curves < 45°.

The percentage reporting pain during pregnancy is comparable to women without AIS and in agreement with results in a large previously published case-control study [6]. Questions about pregnancy and delivery are often raised in AIS. In agreement with previous studies our results indicate that patients can be reassured that scoliosis does not affect pregnancy or delivery.

## Conclusion

We conclude that long-term results were satisfactory in most patients treated with the Boston brace. The primary curve in 86 patients, who had radiological examination at a mean of 19.2 years follow-up, was about the size at the start of bracing and had progressed 5.9° from brace weaning. Six patients had major curve progression. Self-report in 109 patients indicate that future patients can be reassured that scoliosis does not affect pregnancy or delivery. Most patients worked full time. HRQL results were in the normal range in most patients, but the larger variation observed for the scoliosis specific SRS-22 and the use of physiotherapy in about one fourth of the patients, warrant further exploration in future studies.

## Competing interests

The authors declare that they have no competing interests.

## Authors' contributions

JEL designed the study and contributed to collection and interpretation of data as well as manuscript drafting. HS contributed to the design of the study, collection and interpretation of data, and carried out the data analyses and manuscript drafting. JIB contributed to the design of the long-term follow-up, collection and interpretation of data, carried out the data analyses, and wrote the manuscript. All authors read and approved the final manuscript.
